# Supplementary material for: Functional Analysis of the Soybean GmCDPK3 Gene Responding to Drought and Salt Stresses
Source: Int J Mol Sci. 2019 Nov 25;20(23):5909. doi: 10.3390/ijms20235909 (PMC6928923; doi:10.3390/ijms20235909)
Supplement: Supplementary file 1 [file ijms-20-05909-s001.pdf]

Table S1. Details of the 17 GmCDPK proteins. Including sequence ID, protein kinase domain (PKD), Ca^{2+} binding domains (number) (Ca^{2+} BD), amino acid number, predicted molecular weight (MW), chromosomal location and predicted isoelectric point.

**Gene**	**Gene ID**	**PKD**	**Ca^{2+} BD (number)**	**Amino acids**	**MW (kD)**	**Chromosome**	**p*I***
GmCDPK1	Glyma01g37100	S_TKc	EF_hands(4)	562	63.75	1	9.18
GmCDPK2	Glyma02g31490	S_TKc	EF_hands(4)	525	60.04	2	6.2
GmCDPK3	Glyma02g34890	S_TKc	EF_hands(4)	587	65.61	2	6.04
GmCDPK4	Glyma02g48160	S_TKc	EF_hands(4)	549	61.55	2	5.53
GmCDPK5	Glyma03g36240	S_TKc	EF_hands(4)	590	66.54	3	5.15
GmCDPK6	Glyma04g34440	S_TKc	EF_hands(4)	556	62.9	4	6.06
GmCDPK7	Glyma05g01470	S_TKc	EF_hands(4)	539	61.11	5	6.1
GmCDPK8	Glyma05g33240	S_TKc	EF_hands(4)	507	57.07	5	5.25
GmCDPK9	Glyma05g37260	S_TKc	EF_hands(4)	518	58.26	5	5.97
GmCDPK10	Glyma06g20170	S_TKc	EF_hands(4)	551	62.49	6	6.06
GmCDPK11	Glyma10g10501	S_TKc	EF_hands(4)	592	66.23	10	6.12
GmCDPK12	Glyma10g11020	S_TKc	EF_hands(4)	595	65.76	10	5.35
GmCDPK13	Glyma11g08180	S_TKc	EF_hands(4)	562	63.49	11	9.05
GmCDPK14	Glyma14g00320	S_TKc	EF_hands(4)	558	62.73	14	5.51
GmCDPK15	Glyma16g23870	S_TKc	EF_hands(4)	528	59.79	16	8.96
GmCDPK16	Glyma18g11030	S_TKc	EF_hands(4)	551	61.95	18	6.25
GmCDPK17	Glyma19g38890	S_TKc	EF_hands(4)	593	66.96	19	5.43

Table S2. Analysis data of 17 GmCDPK genes for heat map. Expression of 17 GmCDPKs at different tissue and developmental stages was analyzed by SoyBase (https://www.soybase.org/soyseq/)

Gene	Young_ leaf	Flower	One cm pod	Pod shell 10 DAF	Pod shell 14 DAF	Seed 10 DAF	Seed 14 DAF	Seed 21 DAF	Seed 25 DAF	Seed 28 DAF	Seed 35 DAF	Seed 42 DAF	Root	Nodule
GmCDPK1	15	23	12	24	39	17	6	1	17	7	11	3	56	41
GmCDPK2	0	58	2	4	2	2	2	0	0	0	1	3	6	0
GmCDPK3	0	1	0	0	1	0	0	0	0	1	0	0	150	0
GmCDPK4	66	134	77	83	73	52	57	34	76	65	91	43	371	173
GmCDPK5	1	0	0	7	14	2	0	0	0	0	0	0	3	2
GmCDPK6	45	49	37	30	13	11	13	12	13	6	25	11	114	132
GmCDPK7	1	10	1	4	0	3	11	3	6	5	15	5	139	246
GmCDPK8	112	54	104	80	63	21	55	47	80	45	38	32	139	307
GmCDPK9	82	107	106	84	36	23	38	51	70	34	72	65	301	147
GmCDPK10	58	86	65	66	43	21	20	23	30	26	34	23	116	928
GmCDPK11	1	1	0	0	0	0	0	0	0	0	0	0	1	2
GmCDPK12	0	1	0	0	1	3	3	4	2	1	2	0	0	0
GmCDPK13	17	41	31	30	48	15	20	10	14	10	22	2	72	70
GmCDPK14	194	284	178	149	123	72	105	49	136	92	142	100	520	239
GmCDPK15	85	118	88	109	105	57	58	39	99	48	80	56	177	73
GmCDPK16	30	28	29	46	22	5	9	4	25	13	26	12	97	261
GmCDPK17	1	1	1	1	2	0	0	0	0	1	0	1	0	0

Table S3. Distribution and quantity of *cis*-acting elements. To use the PLACE (http://bioinformatics.psb.ugent.be/webtools/plantcare/html/) to identify the cis-acting elements

Gene	ABRE	DRE	ERE	GARE	GT-1	LTRE	MYB	W-box
GmCDPK1	2	—	5	—	1	1	—	—
GmCDPK2	6	—	1	—	1	1	2	—
GmCDPK3	7	—	4	—	2	1	2	4
GmCDPK4	—	—	*2*	—	*3*	—	*2*	*1*
GmCDPK5	7	—	4	—	—	—	1	—
GmCDPK6	—	—	2	—	—	1	—	—
GmCDPK7	1	—	4	1	1	—	—	1
GmCDPK8	1	1	2	—	2	—	4	1
GmCDPK9	1	—	12	—	—	—	3	—
GmCDPK10	3	—	1	—	—	—	3	—
GmCDPK11	4	—	3	—	—	—	3	—
GmCDPK12	3	1	—	—	—	—	1	2
GmCDPK13	4	—	11	—	—	—	1	—
GmCDPK14	3	—	3	—	—	—	4	—
GmCDPK15	1	—	3	—	—	1	3	—
GmCDPK16	—	—	3	—	—	2	2	2
GmCDPK17	5	—	3	—	3	—	3	—

Table S4. Pro, MDA and chlorophyll content of *GmCDPK3*-OE, EV-control and *GmCDPK3*-RNAi transgenic soybean hairy root

	Drought			Salt		
	Pro (μg/g)	MDA (nmol/g)	Chlorophyll (mg/g)	Pro (μg/g)	MDA (nmol/g)	Chlorophyll (mg/g)
GmCDPK3-OE	5.02	35.42	0.64	4.42	31.90	0.83
EV-control	4.88	48.41	0.31	1.91	(41.44	0.77
GmCDPK3-RNAi	4.1	63.76	0.04	1.43	54.57	0.55

Table S5. Primers used in the paper

Gene Primers	Sequences (5'-3')
GmCDPK3 Forward	CTGGAGACAGATCGATAGAG
GmCDPK3 Reverse	ACTAAGTACAACAACCGTGG
GmCDPK3-1302 Forward	GGACTCTTGACCATGGACCTGGAGACAGATCGATAGAG
GmCDPK3-1302 Reverse	GTCAGATCTACCCATGG ACGTACAGGTGGTGCTTCCC
GmCDPK3-GFP Forward	TATCTCTAGAGGATCC CTGGAGACAGATCGATAGAG
GmCDPK3-GFP Reverse	TGCTCACCATGGATCC ACGTACAGGTGGTGCTTCCC
GmCDPK3-3301 Forward	GGACTCTTGACCATG ATGTTTGGAACAAGGAGTAG
GmCDPK3-3301 Reverse	ATTCGAGCTGGTCACC ACGTACAGGTGGTGCTTCCC
GmCDPK1 Forward	CATTATTCCTTTGATCTCTCTCCCA
GmCDPK1 Reverse	TTGCGGTTACGGTTGGATGA
GmCDPK2 Forward	AGAGCCTTGAGGGTGATTGC
GmCDPK2 Reverse	AACCCTACACGCAGCTCATC
GmCDPK3 Forward	AGCTGGTACCACAAAGGGTT
GmCDPK3 Reverse	GTGCTGAGACAAGGGCATCA
GmCDPK4 Forward	GGCAGGAGAGGAGCCAATAC
GmCDPK4 Reverse	TCTCCGACAGATCTCCACAA
GmCDPK5 Forward	TCTTCGTCCATTTTCGTTTCCA
GmCDPK5 Reverse	ATTTCTGCACCAACCCCACT
GmCDPK6 Forward	GCCGGAGAAATCAGAACCCA
GmCDPK6 Reverse	AGGCGTTGCAGTTTCCCATA
GmCDPK7 Forward	GCTGCAGGCCTCAAAATGTC
GmCDPK7 Reverse	TCACACCGCCAATGAGACAA
GmCDPK8 Forward	ATAGCACGCCGAAAAACACG
GmCDPK8 Reverse	GATTTCGCCGCCATTGTTGT
GmCDPK9 Forward	TTGGCTGCAGAGGTACAGAG
GmCDPK9 Reverse	CAACCGCACTTGGGAGACTA
GmCDPK10 Forward	CCGGCGTCACCATACGATAA
GmCDPK10 Reverse	TCTACCACGTCAGCTTTCGC
GmCDPK11 Forward	ACAACTGTGTTGGATCGAGAACT
GmCDPK11 Reverse	TCCGCAGCTTTGGTTCTCTT
GmCDPK12 Forward	AGCTTTGGTTTAATGATGTTTCACT
GmCDPK12 Reverse	GCGGCATTGCATCTCATGATT
GmCDPK13 Forward	TCCTTTGATCTCTCTGCCATTC
GmCDPK13 Reverse	AGACCTTTGCGGTTACGGTT
GmCDPK14 Forward	ACGCCAAGAGAGAAGCCATC
GmCDPK14 Reverse	GGCCCTGTCCCAATTTACGA
GmCDPK15 Forward	GTATCATCCGAACCCCGTCC
GmCDPK15 Reverse	CCTTGCGGTTCTTGTTGACG
GmCDPK16 Forward	ATCTTCCACAATCATCCTTGCAT
GmCDPK16 Reverse	GTGGAGCCTTGAAAGAGGGT
GmCDPK17 Forward	CAACGCGAACAATTCCCACT
GmCDPK17 Reverse	ATGGACGAGGAAAAGAGGTGT
Soybactin Forward	ACATTGTTCTTAGTGGTGGCT
Soybactin Reverse	CTGTTGGAAGGTGCTGAG

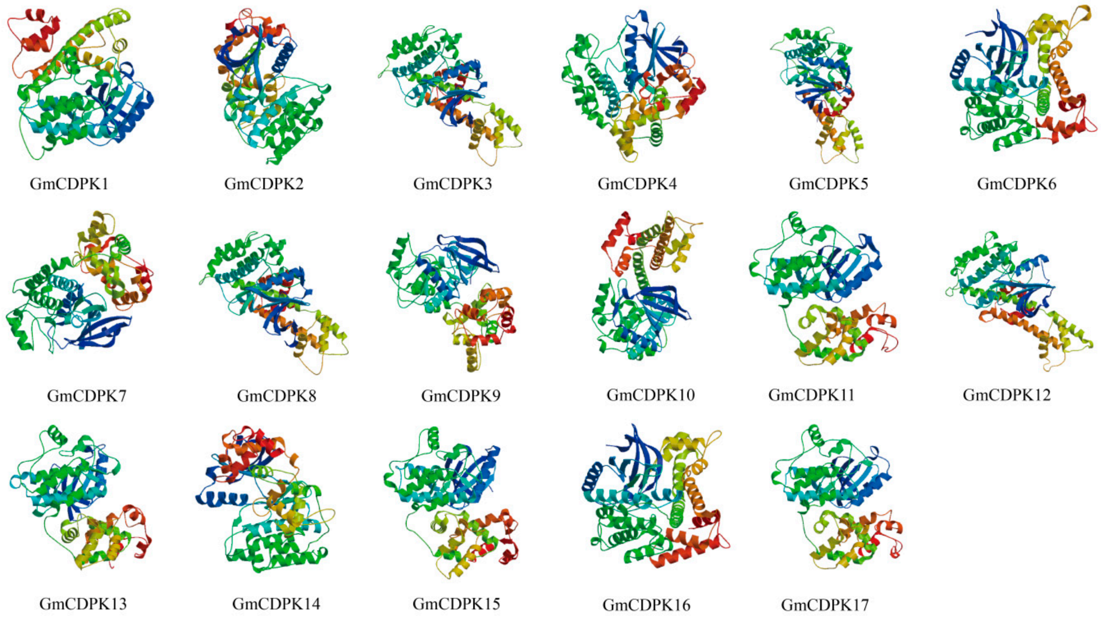

Figure S1. The three-dimensional structure of the protein of 17 GmCDPKs was predicted. The structures of 17 GmCDPKs proteins are shown. Predictions were done using SWISS-MODEL (https://swissmodel.expasy.org/) and evaluated by SAVES (https://servicesn.mbi.ucla.edu/SAVES/), to obtain the predicted structures of soybean CDPK proteins.

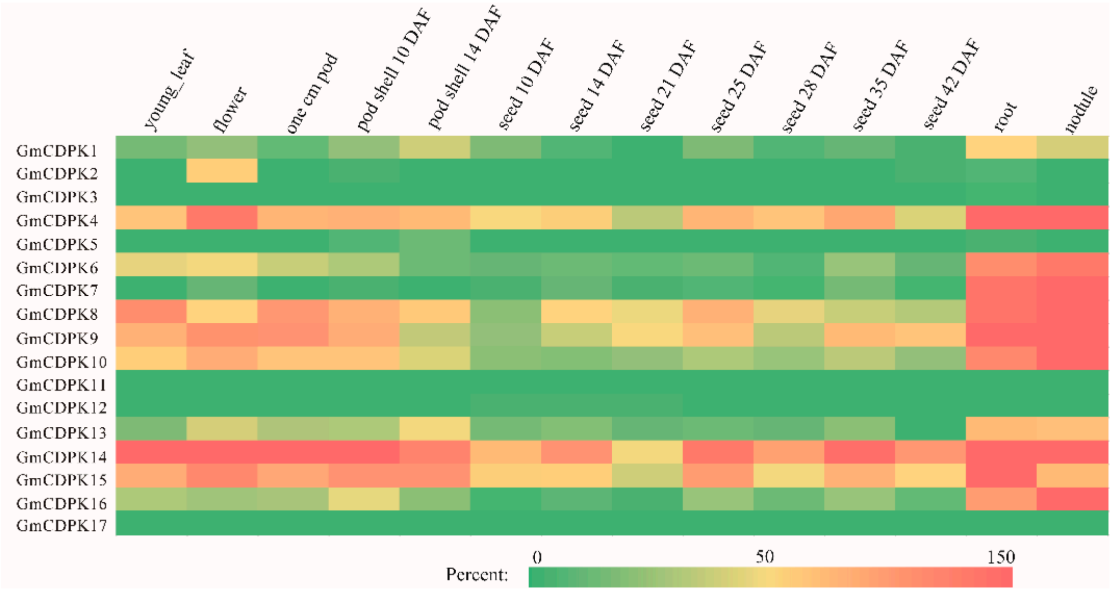

Figure S2. Differential expression analysis of GmCDPKs. To analyze the 17 GmCDPKs in the different tissues and development stages used the SoyBase web (https://www.soybase.org/soyseq/). The heat map drawn with the software EXCEL.

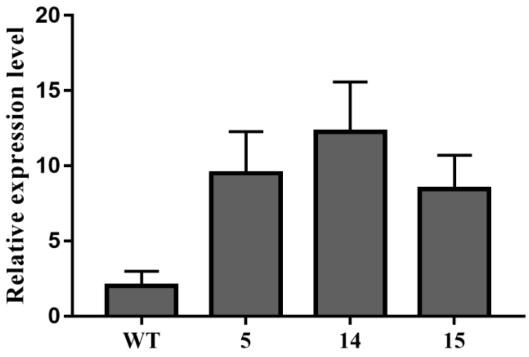

Figure S3. qRT-PCR analyses of GmCDPK3 expression in transgenic Arabidopsis.

厘米

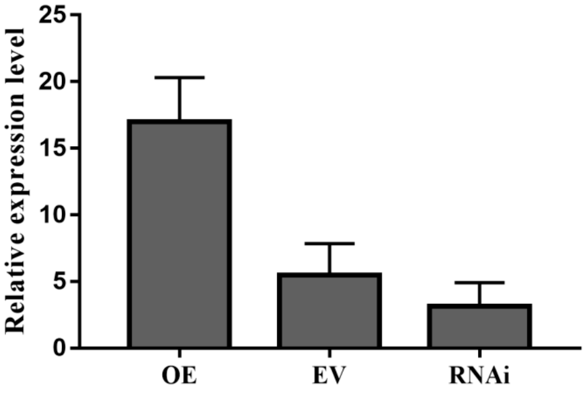

Figure S4. qRT-PCR analyses of *GmCDPK3* expression in *GmCDPK3*-OE, EV-control and *GmCDPK3*-RNAi transgenic soybean hairy root.

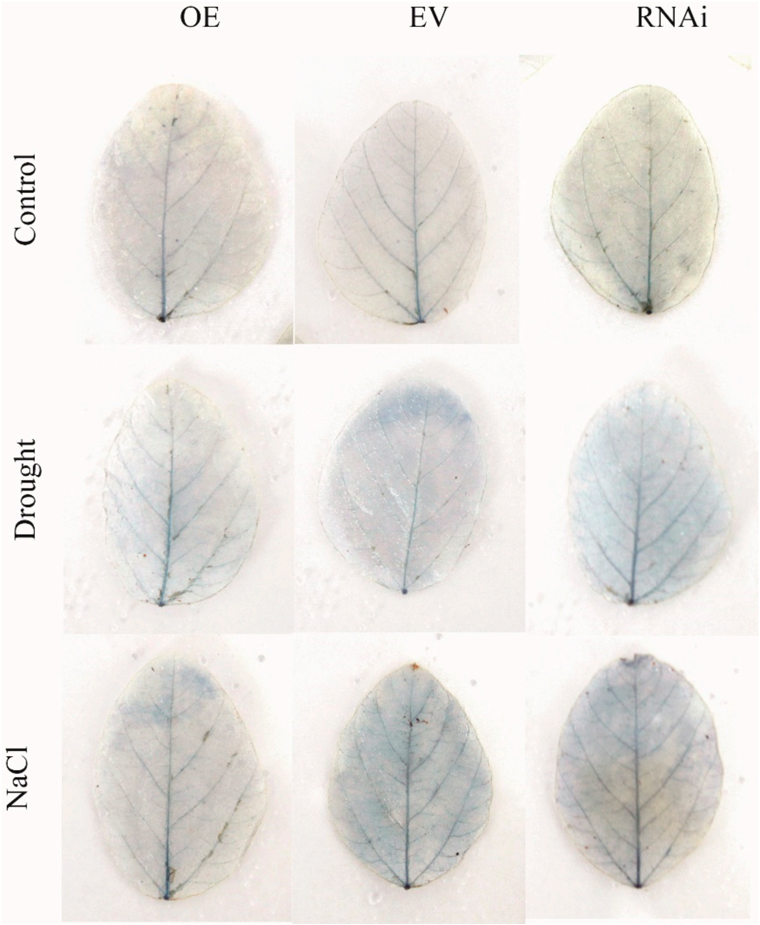

Figure S5. Trypan blue leaves stained of *GmCDPK3* (OE), *GmCDPK3*i (RNAi) and EV. Trypan blue staining under drought and salt stresses.
